# A mechanistic evaluation of the Syrian hamster embryo cell transformation assay (pH 6.7) and molecular events leading to senescence bypass in SHE cells

**DOI:** 10.1016/j.mrgentox.2016.04.002

**Published:** 2016-05

**Authors:** Jessica C. Pickles, Kamala Pant, Lisa A. Mcginty, Hemad Yasaei, Terry Roberts, Andrew D. Scott, Robert F. Newbold

**Affiliations:** a Institute of Cancer Genetics and Pharmacogenomics, Brunel University London, Kingston Lane, Uxbridge, Middlesex UB8 3PH, United Kingdom; b BioReliance Corporation, 14920 Broschart Road, Rockville, MD 20850-3349, USA; c Unilever, Safety and Environmental Assurance Centre, Colworth Science Park, Sharnbrook, Bedford MK44 1LQ, United Kingdom

**Keywords:** SH, syrian hamster, SHE, syrian hamster embryo, CTA, cell transformation assay, Cell transformation assay, Morphological transformation, Senescence bypass, Syrian hamster, p16/CDKN2A

## Abstract

The implementation of the Syrian hamster embryo cell transformation assay (SHE CTA) into test batteries and its relevance in predicting carcinogenicity has been long debated. Despite prevalidation studies to ensure reproducibility and minimise the subjective nature of the assay’s endpoint, an underlying mechanistic and molecular basis supporting morphological transformation (MT) as an indicator of carcinogenesis is still missing. We found that only 20% of benzo(a)pyrene-induced MT clones immortalised suggesting that, alone, the MT phenotype is insufficient for senescence bypass. From a total of 12 B(a)P- immortalised MT lines, inactivating *p53* mutations were identified in 30% of clones, and the majority of these were consistent with the potent carcinogen’s mode of action. Expression of *p16* was commonly silenced or markedly reduced with extensive promoter methylation observed in 45% of MT clones, while *Bmi1* was strongly upregulated in 25% of clones. In instances where secondary events to MT appeared necessary for senescence bypass, as evidenced by a transient cellular crisis, clonal growth correlated with monoallelic deletion of the *CDKN2A/B* locus. The findings further implicate the importance of p16 and p53 pathways in regulating senescence while providing a molecular evaluation of SHE CTA −derived variant MT clones induced by benzo(a)pyrene.

## Highlights

• Mechanistic evaluation of SHE CTA, specific to B(a)P.
• Applicability of the Syrian hamster as a relevant model of carcinogenesis.
• Molecular analysis of immortal lines derived from benzo(a)pyrene-induced MT clones.
• Morphological transformation (MT) does not guarantee senescence bypass.
• Secondary events to MT are necessary for cellular immortalisation.

## 1 Introduction

Reliable assessment of carcinogenic risk is essential for the protection of human health and reducing the incidence of human cancer. Historically, rodent bioassays have been considered the gold standard for toxicological studies, but increasing legislative measures have encouraged the reduction of the numbers of animals used for chemical and agrochemical testing, while placing a complete ban on animal testing in the cosmetics industry [1], [2], [3]. Although a variety of *in-vitro* assays are available for incorporation into test batteries, including those with mutagenicity and chromosomal aberration (clastogenicity) endpoints, cell transformation assays have been considered promising additions given their potential to be able to detect both genotoxic and non-genotoxic carcinogens [4], [5] whilst showing good correlation with rodent bioassay data.

The process of cell transformation is believed to recapitulate stages of carcinogenesis, since fully transformed cells have been shown to possess anchorage-independent growth and to form tumours at sites of injection when explanted into athymic mice [6], [7]. Molecular studies characterising the process of initial events leading to morphological transformation (MT) are limited [8] and have been largely based on the analysis of mass cultured SHE cells treated with agents known to induce transformation [9], [10], even though the SHE-MT assay itself is clonogenic. Despite recent efforts by ECVAM to validate the SHE CTA in terms of assay reproducibility and protocol standardisation while providing photo-catalogues to document colony characteristics [11], [12], [13], the incorporation of the SHE CTA into test batteries has been hindered by concerns over the assay’s subjective nature and lack of available mechanistic data supporting morphological transformation (MT) as a suitable predictor of carcinogenesis.

The Syrian hamster embryo cell transformation assay (SHE CTA) has distinct advantages over other CTAs in that it employs a heterogeneous cell population of normal diploid embryo-derived cells which are capable of metabolically activating carcinogens. This contrasts with the Balb/c and Bhas cells used in other CTAs, which are derived from p53-deficient strains and, in the latter case, express an activated H-*ras* oncogene. Additionally, Syrian hamster cells have a rate of spontaneous immortalisation much more akin to that of human cells, that is to say substantially lower than immortalization frequencies observed in mouse cells [14]. Cellular senescence is an essential barrier against uncontrolled proliferation and must be bypassed during malignant transformation to permit clonal evolution and tumour progression [15], [16]. Human cells reach the end of their proliferative potential due to telomere erosion (known as replicative senescence) which signals a DNA damage response. Senescence can also be induced prematurely, either by oncogene activation (OIS) or as premature stress-induced senescence (SIPS) due to inadequate culture conditions, and in vivo following changes to surrounding microenvironments [17]. In small rodents, telomerase is constitutively switched on meaning that premature senescence can be studied in isolation from events leading to telomere-dependent replicative senescence [18].

The primary objective of the present study was to evaluate further the SHE CTA in order to determine the validity of the MT phenotype in predicting events leading to senescence bypass, and to characterise key somatic genetic and epigenetic events leading to the immortalisation of MT-derived SHE cells, in order to confirm the molecular relevance of the assay to the process of cancer development in vivo.

## 2 Materials and methods

### 2.1 Cell culture

Syrian hamster embryo-derived (SHE) cells were grown at 37 °C ± 1 °C with 10% CO_2_ ± 1% in Dulbecco's modified Eagle's medium LeBoeuf's modification without L-Glutamine (DMEM-L, pH 6.7) (Quality Biological, USA) supplemented with 20% (v/v) fetal calf serum, 1% GlutaMAX™ and 100 units/mL penicillin and 100 μg/mL streptomycin (Invitrogen, Life Technologies, USA). Cells were considered terminally senescent when no signs of cell growth were visible for over a month since seeding and without further sub-culture.

### 2.2 The Syrian hamster cell transformation assay (pH 6.7)

On day 1 primary SHE cells were plated in equilibrated conditioned medium [19] to obtain between 25 to 45 colonies per dish as described by others elsewhere [20]. After 24 h, cells were treated with a final concentration of 5 μg/μl benzo(a)pyrene prepared in a maximum of 0.2% (v/v) DMSO. The final concentration of B(a)P corresponded to that used in positive controls for predictive SHE assays. After a 7 day incubation period cell plates were fixed in methanol and stained in 10% (v/v) Giemsa. Each colony was visually assessed for features of morphological transformation (MT) and scored [12], [21].

### 2.3 Obtaining SHE CTA colony-derived cells

The SHE CTA was performed in parallel at BioReliance (KP) and at Brunel University (JCP). Unstained colonies from treated [B(a)P] and vehicle control (DMSO) plates with clear-cut morphologies (normal or MT) were labelled before returning to the incubator. One plate at a time, the medium was removed and the cells washed in CMF-HBSS (Invitrogen Gibco^®^) before lifting off around half of each colony of interest with a blunted Pasteur pipette. Disaggregated clones were transferred into 0.5 mL DMEM-L conditioned medium (diluted 1:1 with fresh complete DMEM-L) in a 24-well plate to establish colony-derived SHE CTA cultures. The remaining colonies were fixed and stained in Giemsa. At BioReliance, whole colonies were picked using cloning cylinders (KP). From this point onwards clones were expanded until senescence or reaching over 100 PD to confirm immortality.

### 2.4 Gene expression by qRT-PCR

Nucleic acids were extracted and cDNA prepared as previously described [22]. The reference genes *GAPDH* and *beta-actin* were selected from a panel of reference genes (Primerdesign Ltd.) and expression values compared to a panel of six early passage, DMSO-treated SHE cells derived from normal SHE CTA colonies. Primer sequences for qRT-PCR are listed in the supplementary data.

### 2.5 Copy number variation

Separate FAM labelled assays were designed specifically for *p16* (NCBI ref. AH010240.2) exon 1α, *ARF* (AF443796.1) exon 1β and exon 2, *p15* (NM_001281539.1) and *p53* (NM_001281661.1). VIC-labelled *SDHA* (DQ402977.1) was used as the reference gene and duplexed with each gene of interest. 25 μg gDNA was amplified using PrecisionPLUS 2X qPCR Mastermix (Primerdesign Ltd) in a final total volume of 20 μl per reaction each performed in quadruplet and repeated three times. Data was analysed using Copy Caller Software v2.0 (Life Technologies). All primers for CNV were designed and validated by PrimerDesign Ltd. and remain their intellectual property.

### 2.6 Mutation screening

Coding sequences of the tumour suppressor genes *p16* and *p53* were Sanger sequenced using overlapping primers designed to span exon 1α and 2 of *p16* and exons 2–9 of *p53,* as described previously [22] and following gel-excision and purification.

### 2.7 DNA methylation analysis

Colony-derived SHE cultures were analysed for patterns of methylation at the *p16* promoter region. gDNA was bisulphite converted prior to methylation analysis. A 457 bp region containing the SH *p16* promoter was then amplified by PCR using Phusion U polymerase (Thermo Scientific) and gel-excised before insertion into pJET1.2/blunt linearised cloning vector (Thermo Scientific). DNA was extracted from a minimum of 10 bacterial cultures containing the correctly ligated plasmid and bisulphite sequenced to estimate the extent of methylation present at each CpG site. Four MT colony-derived cell lines (BP MT2, BP MT6, BP MT7 and BP MT8) were treated with the demethylating agent 5′-Aza-2′-deoxycytidine and monitored over a period of 4 weeks. Two doses of 5 μM 5-aza-dC (Sigma) were added to fresh culture media at 4 h intervals before removal.

### 2.8 Senescence-associated beta-galactosidase staining

The SA-β gal stain was freshly prepared at pH 6 according to Debacq-Chainiaux et al. [23] in ultra-pure water. Plates were stored at RT in the dark. Fixed and stained cells were imaged and counted for intense blue staining corresponding to SA-β gal activity.

## 3 Results

### 3.1 Morphological transformation does not guarantee senescence bypass

**Fig. 1:**
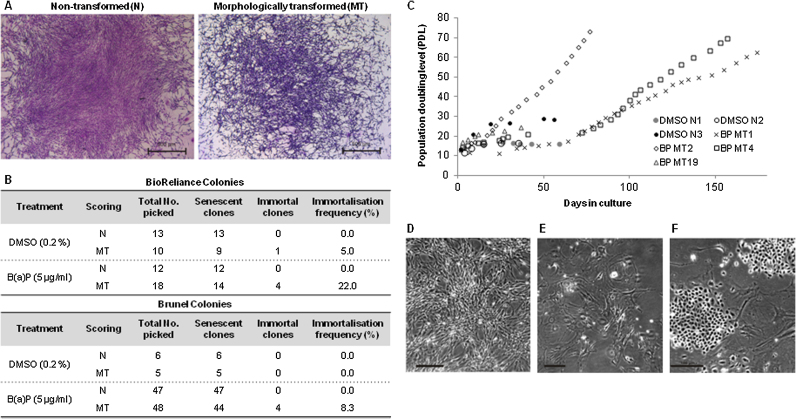
MT characteristics alone are insufficient for cellular immortalisation. The SHE CTA was performed at two laboratories under equivalent conditions with benzo(a)pyrene and DMSO. (A) Examples of normal and morphologically transformed SHE colonies after 7 days growth and stained in Giemsa. (B) Resulting normal and morphologically transformed colonies were picked and grown out to determine cellular lifespan and frequency of immortalisation. (C) Growth curves of picked colonies; the majority of established colonies senescenced by 35 PD. BP MT colony populations that immortalised either continued to proliferate exponentially and retained MT characteristics (D) e.g. BP MT2, or entered a cell crisis following 10–20 population doublings (e.g. BP MT4) noted by increased doubling times and visibly enlarged, senescent-like cells (E). Following a lag period of up to 3 weeks, secondary events subsequent to MT coincided with the appearance of pockets of clonal growth that continued to expand (F).

Normal SHE colonies (Fig. 1A) were well organised, stained light purple in the dibasic Giemsa stain and their cell growth was arranged in a flowing monolayer of contact-inhibited cells, consistent with available photocatalogues [12]. Morphological transformation (MT) was associated with highly disorganised patterns of cell growth along with nuclear aggregation and cell stacking (Fig. 1A). MT cells were typically more elongated with increased nuclear to cytoplasmic ratios and stained dark purple or blue in Giemsa due to their basophilic nature.

To understand the relationship between MT and senescence bypass leading to immortalisation, SHE cells were picked from unstained SHE CTA colonies (supplementary data) and kept in culture to determine their lifespan (Fig. 1B). Irrespective of treatment and laboratory performing the CTA, all normal colony-derived cells ceased to proliferate before 35 population doublings (Fig. 1B and C) and entered senescence as judged by increased SA-β gal staining, general cell enlargement and flattening (data not shown). Cells were maintained in this senescent state with no signs of cell division for up to two months. Cells derived from MT colonies also mostly entered terminal senescence by 35 PD (Fig. 1B and C), indicating that scoring of MT alone is insufficient to guarantee senescence bypass. We observed an immortalisation frequency of 20% and 8% for BP MT colonies picked at the BioReliance and Brunel laboratories respectively (Fig. 1B).

### 3.2 Secondary event(s) to MT characteristics permit immortalisation

A total of 10 B(a)P – treated MT colony-derived cultures acquired immortality and continued to proliferate beyond 100 PD from the single cell stage; 4 clones originating from BioReliance and 6 from Brunel. Half of the immortalised BP MT colonies were found to be immortal from the outset and continued to exponentially expand with 22–33 h population doubling times (Fig. 1C). The remaining BP MT colony-derived cultures that went on to immortalise, entered a cellular crisis characterised by increased population doubling times of up to 80 h, that lasted around 20 days (Fig. 1C). During this temporary phase SHE cells became senescence-like in terms of cell morphology (Fig. 1E) before the emergence of rare pockets of clonal growth (Fig. 1F) which continued to proliferate indefinitely. A single rare DMSO-treated MT scored colony gave rise to a population of cells with indefinite growth potential, also emerging after a prolonged period of cellular crisis, suggesting a spontaneous frequency of immortalisation equal to 0–5% MT colonies.

We conclude that the MT phenotype and endpoint of the SHE CTA does not guarantee evasion of senescence barriers but may predispose SHE cells to acquiring cellular immortality following secondary rare and necessary events for senescence bypass. Such events are clearly observable following colony picking and establishment at around 20 PD due to the drastic changes in cell morphology as the majority of the cell population enters senescence. In contrast, in instances where no crisis period was observed, SHE BP MT clones appeared immortal from the outset although secondary events to MT may have still taken place early on during colony formation and thus not observable in terms of cell morphology and division rates.

### 3.3 P53 mutations present in 30% of immortal SHE MT colony-derived clones

Our previous studies using Syrian hamster dermal cells (SHD) indicated that senescence bypass induced by benzo(a)pyrene could be achieved by a co-operative two-step mechanism involving *p53* transversion mutations and *p16* transcriptional gene silencing [22]. Colony-derived SHE cells were screened for mutations in the coding regions of the key tumour suppressor genes *p53* and *p16* (AF292567). No mutations were identified in *p16* sequences, although c345CT > TC was common to all cultures and controls tested and matches the newly sequenced genomic information for *Mesocricetus auratus* (WGS Project APMT01). Similarly, a common variant was also identified for *p53* (c561 GA > AG), as shown in Table 1. A further two SHE CTA-derived B(a)P-treated MT lines (BP MT11 and BP MT12) were sourced from previous studies in our laboratory [24] bringing the total BP MT-immortalised cultures to twelve. *p53* point mutations were identified in four separate BP MT lines; all were located within the protein’s DNA binding domain corresponding to known human *p53* mutational hotspots (Table 1), predicted to negatively impact p53 protein function [25] and largely consistent with transversion mutations typical of B(a)P’s well-characterised mode of action. Thus 30% of immortalised B(a)P-treated lines MT lines were found to have a mutated *p53* whilst no point mutations were identified in any normal (N) colony-derived cells or in the spontaneous DMSO MT line. Analysis of *p53* mutants at early population doublings, coinciding with when the cultures were initially established, suggests a heterogeneous cell population indicating that the presence of *p53* point mutations conferred a selective growth advantage.

### 3.4 Reduced expression of CDKN2A/B genes in immortal SHE MT colony-derived cells

**Fig. 2:**
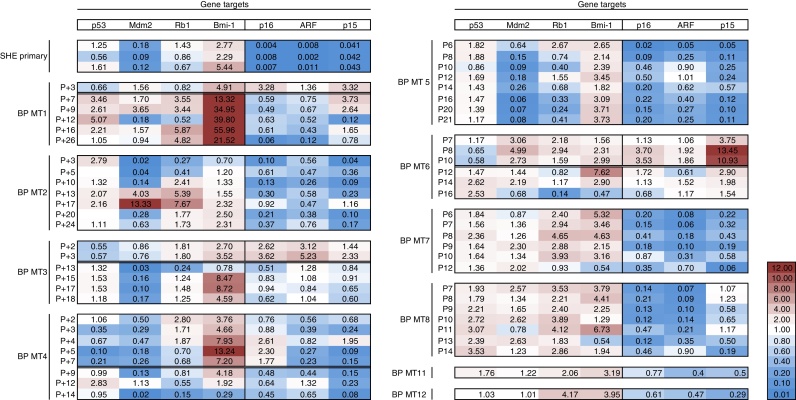
Gene expression in SHE CTA derived B(a)P-induced clones. ‘Heat map’ summarising gene expression profiles for p16 and p53-pathway members in immortalised B(a)P-induced SHE CTA cell lines at successive passages during their lifespan. Ct values were normalised to the reference genes GAPDH and beta-actin and compared to DMSO-treated, early passage non-transformed colonies derived from the SHE CTA. The double strikethrough line indicates the time point at which a crisis-phase was observed across the cell population and senescence barriers were overcome.

Expression of senescence-associated genes was analysed in BP MT colony-derived cells as the cells bypassed senescence to identify any common characteristics and potential predictive biomarkers in MT clones. Levels of *p16* transcripts were generally reduced in immortalised B(a)P-induced clones and *ARF* and *p15* were also expressed at low levels (Fig. 2). In contrast *p16* was overexpressed in senescent SHE cells derived from DMSO non-transformed colonies, while primary untreated SHE cells minimally expressed all three *CDKN2A/B* transcripts. An increase in the expression of *p16* and *p15* was associated with the observed increase in senescent-like morphology and increase in population doubling times in clones BP MT1, BP MT3, BP MT4 and BP MT6 (Figs. 1 C and 2). Transcript levels of *p16* and *p15* were markedly decreased in emerging immortal cells which went to on divide indefinitely. *ARF* was also generally repressed but the differences in transcript abundance appeared to be more subtle pre- and post-crisis. The simultaneous change in transcript levels and cell morphology indicates the importance of functional *p16* and also *p15* in promoting growth arrest and suggests inactivation of these senescence pathways during immortalisation.

### 3.5 Over-expression of bmi1 mRNA in immortal SHE MT colony-derived cells

*Bmi-1* expression was quantified in our various SHE cell clones. This analysis indicated a strong overexpression of *Bmi1* transcripts, especially in BP MT1 (Fig. 2). Given that Bmi1 has been shown to act oncogenically and has been implicated in inducing immortalisation [28], we hypothesised that its over-expression may have led to *p16* downregulation and contributed towards senescence bypass in SHE clones. Further work is needed to confirm this regulatory phenomenon in Syrian hamster cells. In human cells, Bmi1 is known to regulate *p16* transcription both by direct binding to an upstream gene element and indirectly at a chromatin level by Polycomb protein assembly [29], [30].

### 3.6 Single copy loss of CDKN2A/B genes

In an effort to explain further the mechanism of senescence bypass in SHE CTA colony-derived MT lines, separate copy number variation assays were carried out for the three genes encoded at the *CDKN2A/B* locus and for *p53*. All colony-derived cells retained 2 copies of *p53,* but single allelic loss was observed within the *CDKN2A/B* locus in 5 out of 12 immortal BP MT colony-derived cultures (Table 2). Interestingly 3 out of 5 BP-induced immortalised lines that overcame a cell crisis phase (with prolonged population doubling times) were found to have lost a copy of the *CDKN2A/B* locus. BP MT1 and BP MT6 had a single copy of the entire *CDKN2A/B* locus encompassing *p16, ARF* and *p15* but when analysed prior to 35 population doublings both copies were present, suggesting the deletion of one allele during the immortalisation process. The remaining 2 lines were predicted to have two copies of the *CDKN2A/B* locus, with increased doubling times associated with the accumulation of its *p53* mutation (discussed earlier). Similarly, the entire *CDKN2A* locus was found to be monoallelic in BP MT11 and BP MT12 while BP MT3 lacked a copy of exon 2, which is common to both *ARF* and *p16,* as well as exon 1β which is unique to *ARF* (Table 2). Collectively, these findings further suggest that loss of key tumour suppressor genes at the *CDKN2A/B* locus contributes towards senescence bypass and, additionally, may point towards haploinsufficiency contributing to abrogation of senescence pathways.

### 3.7 DNA methylation of the SH p16 gene promoter in SHE MT colony-derived immortal cells

**Fig. 3:**
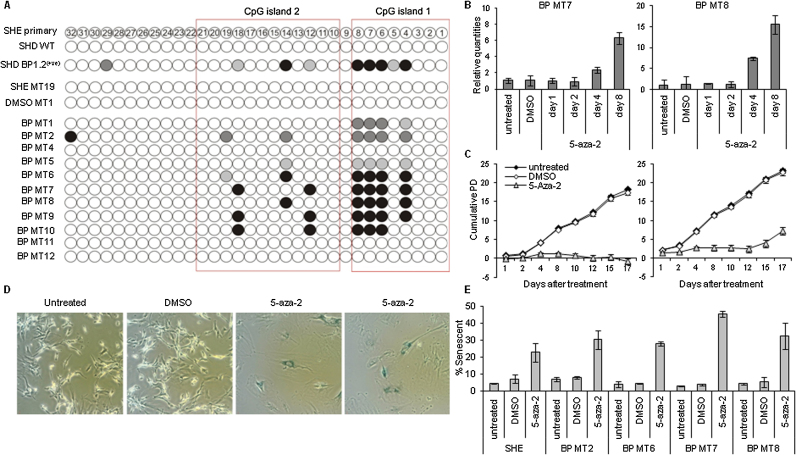
The *p16* promoter is methylated in 45% B(a)P-induced immortal MT clones. Bisulphite sequencing was performed on the p16 promoter region of B(a)P-induced immortal MT colony-derived SHE cell lines. The extent of DNA methylation was analysed at 32CpG sites; CpG site 1 is closest to the ATG start site and CpG site 32 is the most distant. A minimum of 10 colonies per sample were analysed. (A) 45% of analysed MT colonies were heavily methylated (black dots) and a further 25% of MT colonies were partially methylated at similar CpG sites. White (empty) symbols indicate no methylation, black symbols represent >50% methylation, dark grey symbols 30–40% and light grey symbols 20% at each CpG site. When treated with 5-aza-dC methyl groups were removed following 4–8 days in BP MT2, BP MT7 and BP MT8 (supplementary) and corresponded to increases in p16 gene expression (B). (C) Treated cells were counted every 2–3 days to calculate cumulative population doublings. (D) After 10 days of treatment plates were stained for SA-β gal as an indicator of cellular senescence; (E) the percentage of SA-β gal positive cells increased due to 5-aza-dC treatment. A minimum of 100 cells were counted per plate in triplicate, error bars represent the standard deviation from the mean.

Our recent studies using carcinogen-induced SH dermal cell clones indicated that silencing of *p16* gene expression was associated with promoter methylation [22]. In order to provide a more comprehensive explanation for the reduced *p16* transcript levels in SHE CTA immortal colony-derived cultures, DNA methylation levels were analysed in a 450 bp region upstream of the *p16* start site. Bisulphite sequencing showed that over 45% of all immortal MT colony-derived SHE cells had methylated *p16* promoters, with methyl groups commonly located within the identified CpG islands [31] (Fig. 3A). Patterns of promoter methylation were also observed using methyl-specific PCR (data not shown). No methylation was observed in any of the controls or in the spontaneous line DMSO MT1.

To confirm that *p16* gene expression could be regulated by promoter methylation, BP MT lines were treated with the methyl-transferase inhibitor 5-aza-2′ deoxycytidine (5-aza-dC). From 48 h following the initiation of 5-aza-dC treatment, the proliferation rates of the cells decreased (Fig. 3C), while the percentage of senescent cells increased as shown by SA-βgal staining and domination of the cultures with morphologically senescent cells (Fig. 3D and E). After 4–8 days, transcript levels of *p16* significantly increased compared to untreated cells (Fig. 3B) and methyl groups were found to have been lost (Supplementary data). In the case of BP MT7, treatment with the demethylating agent appeared to have permanent effects; the cells remained senescent and ceased to show signs of proliferation (Fig. 3C). All other treated lines started to recover 2 weeks after the addition of 5-aza-dC, coupled with a return to low *p16* expression levels. The data therefore provide persuasive evidence that aberrant DNA methylation can also contribute towards senescence-bypass of SHE CTA derived cultures.

## 4 Discussion

The merits of incorporating the SHE CTA into test batteries for safety and toxicological testing have long been debated. Although recent efforts by ECVAM to pre-validate the assay in terms of reproducibility [13], [21], [32] and by the OECD to provide standardised assay guidelines, are well advanced [33], a lack of molecular understanding forging a mechanistic link between the assay’s end point and carcinogenesis has hindered its widespread adoption as a suitable animal alternative [3]. Here, we have further evaluated the SHE CTA by picking representative examples of MT and non-transformed colonies prior to staining in Giemsa and characterising molecular events leading to unlimited growth potential in cells derived from benzo(a)pyrene-induced morphologically transformed (MT) clones.

We find that MT is insufficient for senescence bypass. The vast majority of MT clones ceased to proliferate before they had undergone 35 population doublings. Only 10–20% of B(a)P-treated MT-derived clones continued towards immortality which was not dissimilar to previous SHE growth studies where fewer than 30% of MT colonies were estimated to immortalise [34]. Events secondary to the MT phenotype were often necessary for senescence bypass, as indicated by the clear-cut crisis phase observed in around half of the established SHE lines after 2–3 months following colony picking. Rare events gave rise to rapidly dividing cells, which curiously had lost their MT phenotype, an observation that has been noted previously by others. Additional events have historically been linked to cells acquiring the ability to grow in soft agar (anchorage-independence) and to form tumours at the site of injection in athymic mice [34], [35]. This is consistent with the notion that immortalisation and senescence bypass, while rate-limiting, are only the first necessary steps towards progression to malignancy [14], [36], [37].

Mutational analysis of *p53* revealed that 30% of immortalised B(a)P-induced MT lines contained likely deleterious missense mutations in the tumour suppressor gene DNA binding domain [25], [27]. The expected mutation frequency of B(a)P for a given haploid gene (eg *hprt*) in Chinese hamster cells is 3 × 10^−4^/treated cell, which is approximately thirty times greater than the spontaneous rate of mutation [38]. Our observation that a relatively high proportion of immortal SHE MT clones contained a *p53* mutation, can be explained by the prolonged 7-day incubation of SHE cells initially exposed to B(a)P during the SHE CTA. At the end of the assay the average SHE colony contained 8 × 10^3^ cells and was thus estimated to undergo around 13 population doublings in the presence of the carcinogen. Precisely how heterogeneous the embryo cells are within a given colony and how this affects the population’s growth potential remains to be established. The data would suggest that those cells containing a *p53* mutation were positively selected during successive subcultures as, post crisis, all transcripts analysed contained the mutated sequence.

Immortal MT colony-derived cell lines that emerged from a senescence-like phase without known point mutations in *p53* possessed only single copies of the genes encoded by the *CDKN2A/B* locus. Gene expression analysis associates the sudden decrease in *p16* and *p15* transcripts with copy number loss following the emergence of rare clonal growth. Down-regulation of *p16* and *p15* mRNA expression at subsequent time points analysed remained stable, reflecting the permanent allele loss. Deletions spanning 9p21 in humans have been commonly associated with melanoma and multiple other tumour types [39], [40] while copy number loss in the Syrian hamster has previously been described in pancreatic and oral tumours [41] and in our own laboratory has recently been associated with carcinogen-induced senescence-bypass [22].

DNA methylation of several specific CpG sites located within the first 300 bp upstream of the *p16* promoter was observed in BP MT-immortalised colony-derived cells. 40% of BP MT clones were heavily methylated at CpG sites 40 bp upstream of the TSS, while presence of methylated CpG dinucleotides were observed in another 27% of clones at the same residues, suggesting a regulatory role. A putative TATA box was identified at −160 bp to the transcription start site (data not shown) and three more commonly methylated CpG sites were identified upstream of this. Although most BP MT colony-derived cells expressed *p16* at low levels, lines expressing the lowest levels of *p16* were identified to have the highest extent of DNA methylation at the *p16* gene promoter. This is consistent with an inverse relationship in SH cells between the level of *p16* transcripts and presence of methyl groups [42], [43], although promoter methylation status alone was not sufficient to predict senescence bypass. Treatment with the methyltransferase inhibitor 5-aza-dC led to a significant increase in *p16* expression after four and eight days exposure to the drug and this was accompanied with the temporary loss of methyl groups at the *p16* promoter, a decrease in cell division rates, and an increase in senescent cells detected by SA-βgal staining. The data suggests that DNA methylation at the promoter is important in suppressing *p16* transcription and is likely to have a co-operative role in evading senescence barriers since, overall, almost 70% of B(a)P-induced MT-derived lines were methylated.

Our previous studies using SH dermal fibroblasts and a mass culture approach, have characterised carcinogen specific fingerprints reflecting their mode of action, leading to senescence bypass and cellular immortalisation. A two-step model has been proposed for B(a)P-induced SHD immortalisation: *p53* inactivation by point mutations (mainly G to T transversions) initially, followed by a ‘second hit’ targeting the p16-Rb pathway, either by epigenetic silencing of *p16* by DNA methylation, *p16* single copy loss, or *p15* and *Rb1* transcriptional down-regulation [22]. By comparison, in the present study, SHE immortal clones were overall found to have low levels of *p16* and 70% of clones did not contain *p16/ARF* or *p53* mutations. Extensive promoter methylation and/or *CDKN2A/B* locus single allele loss were correlated positively with the reduced transcript levels identified. Additionally, we hypothesise that overexpression of PRC1 polycomb protein Bmi-1 may have contributed towards *p16* repression and evasion of senescence barriers. Whether senescence in these instances was bypassed solely via the p16/pRB pathway in isolation from p53 signalling is unknown given that we were unable accurately to quantify levels of the p53 and p16 proteins via western blot (due to a lack of available antibodies to SH). Although the p53 qPCR data suggests no large change in gene expression, we cannot exclude that aberrant post transcriptional modifications and/or signalling of downstream effectors such as p21 are also likely to have played a role in evading senescence barriers.

The molecular analysis of SHE CTA derived clones described here provides additional evidence supporting the applicability of the Syrian hamster as a suitable model for carcinogenesis studies. Along with our earlier studies using SHD cells [22 INK4B and p53 tumour-suppressor genes drive induced senescence bypass in normal diploid mammalian cells] it underscores its value in gaining an understanding of senescence bypass mechanisms that drive SIPS and OIS in isolation from the telomere-mediated replicative senescence barriers that predominate in human cells [14], [18]. In the past the lack of sequence information for the Syrian hamster has been experimentally restrictive for pursuing such studies [3], [22]. However, the *Mesocricetus auratus* genome has now been shotgun sequenced and assembled into unannotated contigs (WGS Project APMT01) and even more recently, its transcriptome has been released [44] thanks to the use of SH in modelling Ebola [45]. Such ‘-omics’ advances will ultimately be beneficial in the implementation of SH as a suitable model. As an example, our sequence alignments of coding regions to WGS sequences (see supplementary data) suggest that the *CDKN2A/B* locus is well conserved in SH, of a similar size to that in humans (i.e. in excess of 35 KB) with the same genomic organisation including the upstream regulatory domain (RD) [41].

Efforts to improve and/or automate the scoring process of the SHE CTA have had limited success. Such developments include computerised image analysis to detect alterations in colony colour, organisation and texture [46], and use of attenuated total reflection Fourier-transform infrared (ATR-FTIR) microscopy to identify biochemical signatures of MT [47], [48], [49]. No unique predictive attribute was visually identified from the remaining colony portions imaged that in culture had unlimited growth potential. In our present study, by picking and growing out morphologically transformed colonies it was possible to uncover some of the key molecular events leading to immortalisation of MT clones.

We appreciate that the data presented here is limited to B(a)P-induced effects, a known potent mutagen and carcinogen commonly used as one of the SHE CTA positive controls. We recommend that the analysis now be extended to other known carcinogens, including those inducing epigenetic or non-genotoxic events, as proof of principle. Final concentrations of B(a)P used in this study (5 μg/ul) are estimated to be saturating in Syrian hamster primary cells, as levels of DNA binding by its highly mutagenic metabolised products [primarily 7,8-diol-9,10-epoxide-B(a)P (BPDE)] level off at around a fifth of this dose [50]. It may therefore be worth considering how the observed molecular events may change in relation to a decrease in chemical exposure. Given that the morphologies of SHE MT colonies differ slightly when performed at pH 6.7 compared to pH 7.1–7.3 [21] we are currently unable to comment on whether senescence bypass and subsequent pathway aberrations take place at similar frequencies between all versions of the SHE assay.

In conclusion, we provide an underlying mechanistic analysis of the SHE CTA, as performed at pH 6.7. Ultimately, if combined with suitable mechanistic and molecular data, the SHE assay could provide a suitable *in vitro* predictor of carcinogenic potential to be incorporated into regulatory testing strategies. In addition, the implementation of (Q)SAR modelling alongside cell transformation assays has been proposed to address false negatives and false positives in order to add further towards a weight of evidence approach when assessing chemical data [51]. Of critical importance, the SHE CTA has scope for both detecting and determining the mode of action of non-genotoxic carcinogens, which typically cannot be reliably identified by standard mutagenicity testing alone.

## Figures and Tables

**Table 1:** *p53* point mutations confer growth advantages and are identified in B(a)P-induced clones but no *p16* mutants were identified.

	p16	Codon change	Translated mutation		Human equivalent	Hotspot	Location
All	c345CT > TC	CAC/TGC > CAT/CGC	115–116aa	HIS/CYS > HIS/ARG	116aa HIS/ARG	0	Exon 2

	p53	Codon change	Translated mutation		Human equivalent	Hotspot^a^	Location
All	c561 GA > AG	GAG/AGC > GAA/GGC	187–188aa	GLU/SER > GLU/GLY	185aa ASP/SER	18 (0)	DBD
BP MT9	c482 G > T	CGT > CTT	161aa	ARG > LEU	158aa ARG	264 (102)	DBD
BP MT10	c808C > T	CGG > TGG	270aa	ARG > TRP	267aa ARG	65 (34)	DBD
BP MT11	c734 G > C	TGC > TCC	245aa	CYS > SER	242aa CYS	198 (20)	DBD
BP MT12	c752 G > T	CGG > CTG	251aa	ARG > LEU	248aa ARG	1544 (121)	DBD

Colony-derived SHE CTA cells were screened for *p53* and *p16* mutations within gene coding regions. (A) No p16 point mutations were identified, (B) four non-synonymous *p53* mutants were identified in established B(a)P-treated, MT colony-derived SHE lines all located within the DNA binding domain (DBD); numbers in brackets represent the number of known human tumours with the same amino-acid mutation according to IARC *p53* database [27].

a Number of human *p53* mutations found at that codon according to the Universal mutation database (UMD) [26].

**Table 2:** Summary of genetic changes in SHE CTA colony-derived cells.

	Immortal	Cell crisis	p53 mutation	p16-methylation	Gene copy number (CNV)				
					p16 exon1α	p16/ARF exon2	ARF exon1β	p15	p53
SHE primary	N								
DMSO N	N								
DMSO MT1	Y	Y							
BP N	N								
BP MT1	Y	Y		Y	−/+		−/+	−/+	
BP MT2	Y			Y					
BP MT3	Y	Y				−/+	−/+		
BP MT4	Y	Y							
BP MT5	Y			Y					
BP MT6	Y	Y		Y	−/+	−/+	−/+	−/+	
BP MT7	Y			Y					
BP MT8	Y			Y					
BP MT9	Y		p.R161L	Y					
BP MT10	Y	Y	p.R270W	Y					
BP MT11	Y	n/a^a^	p.C425S		−/+	−/+	−/+	−/+	
BPMT12	Y	n/a^a^	p.R251L		−/+	−/+	−/+	−/+	

SHE CTA colony-derived cells were analysed for gene mutations in *p16* and *p53*, DNA methylation at the *p16* promoter and copy number variations across the CDKN2A/B locus and *p53*.

a Unknown.
