# Supplementary material for: A mechanistic evaluation of the Syrian hamster embryo cell transformation assay (pH 6.7) and molecular events leading to senescence bypass in SHE cells
Source: Mutat Res Genet Toxicol Environ Mutagen. 2016 May;802:50–8. doi: 10.1016/j.mrgentox.2016.04.002 (PMC4877681; doi:10.1016/j.mrgentox.2016.04.002)
Supplement: Supplementary file 1 [file mmc1.pdf]

Supplementary figures

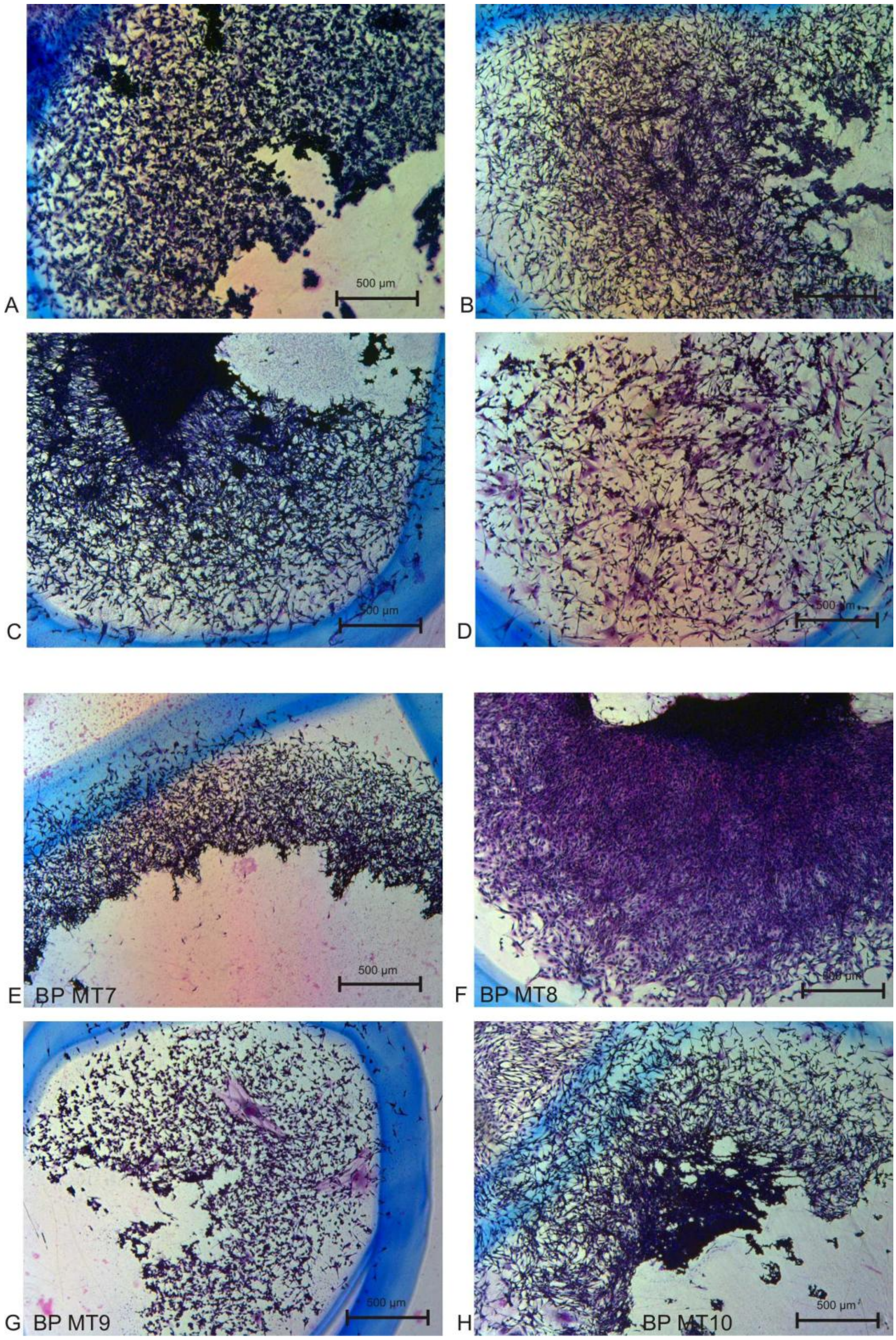

Supplementary Figure 1. Picking morphologically transformed colonies

In order to establish cultures derived from SHE CTA colonies, cells were lifted off from SHE CTA colonies prior to staining and their scoring confirmed by subsequent staining in Giemsa. (A-D) BP MT colonies that stopped growing following plating and (E-F) BP MT colonies that continued to proliferate beyond 100 PD and can be considered immortal, established lines labelled. No common characteristics were observed for MT colonies acquiring unlimited lifespans.

Supplementary figures

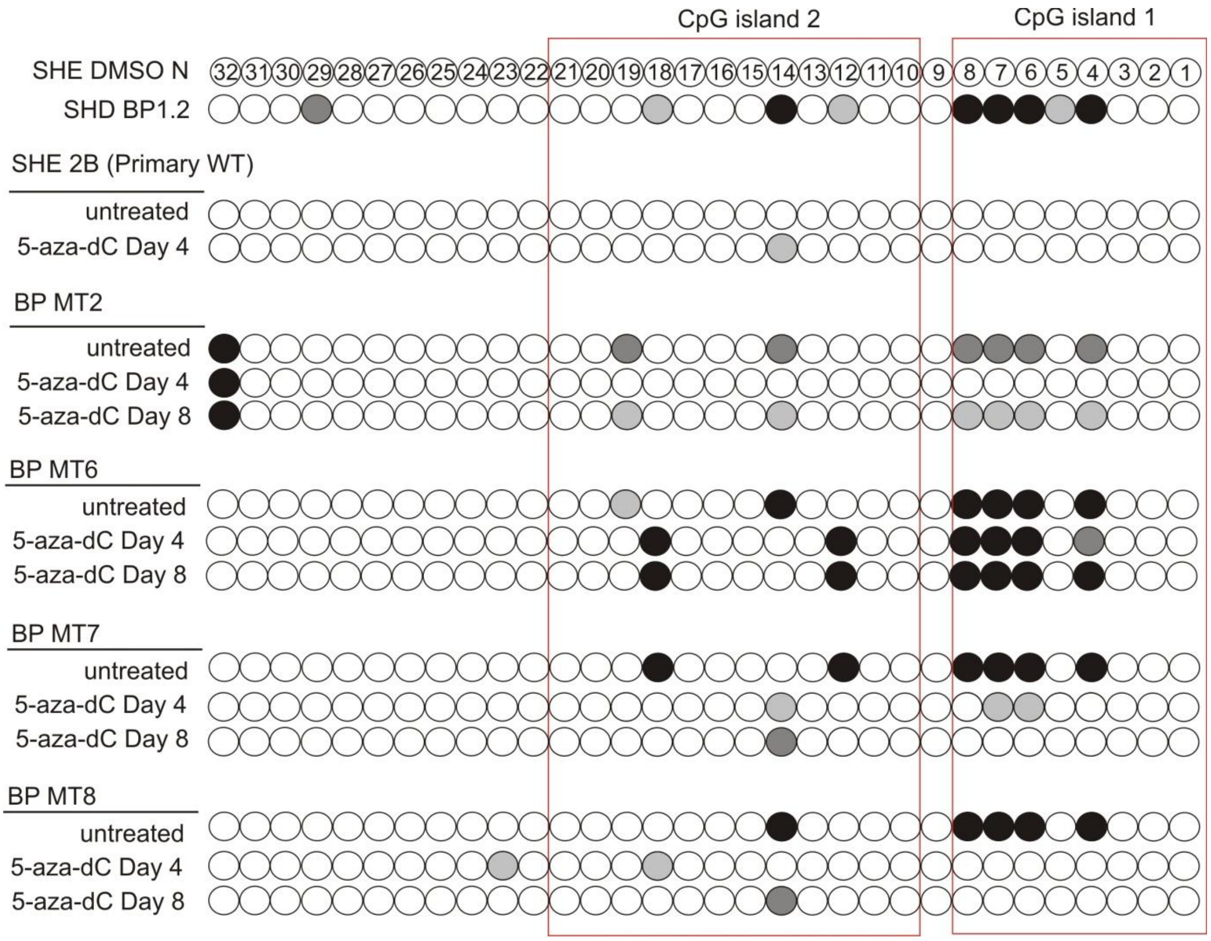

Supplementary Figure 2. Removal of methyl groups at the p16 promoter following exposure to 5-aza-dC after 4 and 8 days

Cells were treated with the methyltransferase inhibitor 5-aza-dC and DNA was extracted from cells 4 and 8 days after exposure. Bisulphite converted gDNA was then amplified using bisulphite sequencing primers and *p16* 5'-promoter sequences were analysed for DNA methylation at 32 CpG sites; CpG site 1 is closest to the ATG start site and CpG site 32 is the most distant. A minimum of 10 colonies per sample were analysed. White (empty) symbols indicate no methylation, black symbols represent >50 % methylation, dark grey symbols represent methylation at 30-40 % of samples and light grey symbols in 20 % of samples at each CpG site.

Supplementary figures

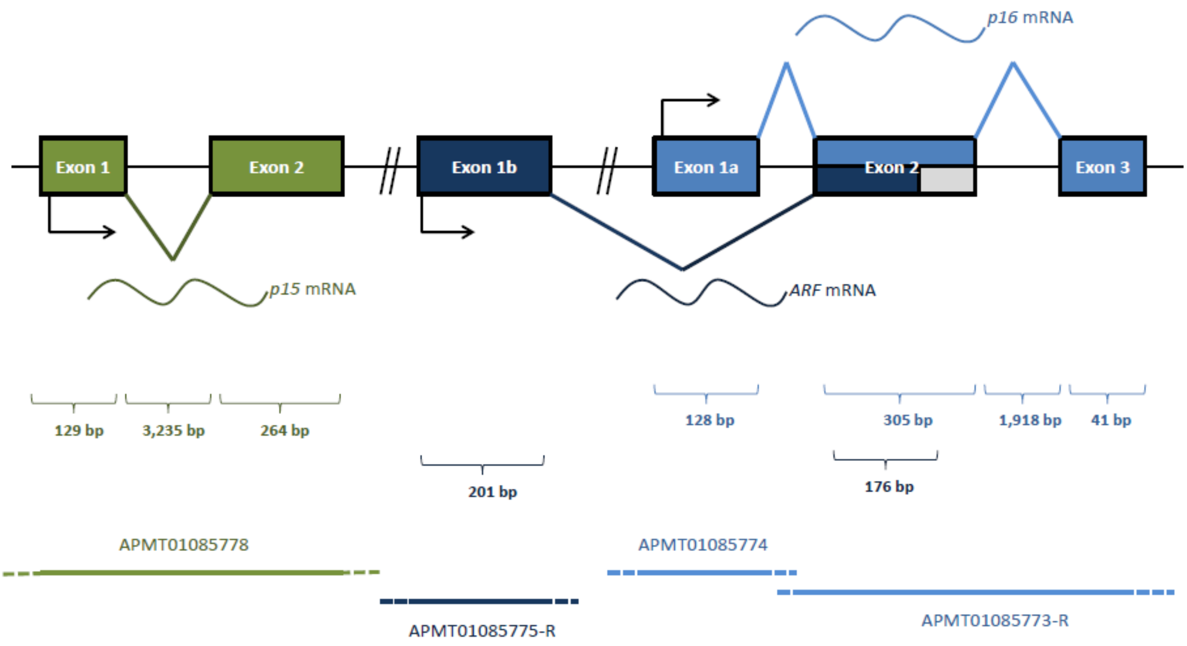

Supplementary Figure 3. Suggested *CDKN2A/B* locus in *Mesocricetus auratus*

Gene transcripts of the *CDKN2A/B* locus in the Syrian hamster mapped to 4 different WGS contigs. Coding sequences for *p16*, *p15* and *ARF* were aligned to WGS available via the NCBI nucleotide database using CLC sequence viewer v6.9. The number of exons in each gene was comparable to their human counterparts and the alignments suggested a very similar genomic layout of *p16*, *p15* and *ARF*. The figure is not to scale.
