# Supplementary material for: A mechanistic evaluation of the Syrian hamster embryo cell transformation assay (pH 6.7) and molecular events leading to senescence bypass in SHE cells
Source: Mutat Res Genet Toxicol Environ Mutagen. 2016 May;802:50–8. doi: 10.1016/j.mrgentox.2016.04.002 (PMC4877681; doi:10.1016/j.mrgentox.2016.04.002)
Supplement: Supplementary file 2 [file mmc2.docx]

**Supplementary Table 1:** **Primer sequence list**

| Gene expression real time PCR primers  ***GAPDH***  5’-TTGTTGCCATCAATGACCCCTT-3’(forward)  5’-CGTTCTCAGCCTTGACTGTGCCTT-3’ (reverse)  ***Beta Actin***  5’-ATGGCCAGGTCATCACCATT-3’(forward)  5’-TGTAGTTTCGTGGATGCCACA-3’ (reverse)  ***p16 (Cdkn2a)***  5’- agaggttcgggctttgct-3’ (forward)  5’-CTACTTGGGTGTTGCCCATC-3’ (reverse)  ***ARF***  5’-GCAGGTTCGTGGTGACTGT-3’ (forward)  5’-CTCGCTAGCATCAACAGCAG-3’ (reverse)  ***p53***  5’-CCCCCAAAGAGTGCTAAACGA-3’ (forward)  5’-CAGTTCCAAGGCCTCATTCAA-3’ (reverse)  ***p15 (Cdkn2b)***  5’-CTGTGAGAGGAGGACAAGGG-3’ (forward)  5’-CATCATCATGACCTGGATCG-3’ (reverse)  ***Mdm2***  5’-CACAGGTCCCTTTCCTTTGA-3’ (forward)  5’-TGAATCCTGATCCAGCCAAT-3’ (reverse)  ***Rb1***  5’-CGCCTTCTGTCTGATCATCCA-3’ (forward)  5’-TTGGTCCAAAT GCCGGTCT-3’ (reverse)  ***Bmi1***  5’-CTGGAGAAGAAATGGCCCTCT-3’ (forward)  5’-TTCTCCCGCATTTGTCAGC-3’ (reverse)  Sequencing primers  ***p53***  Exons 2-4  5’-GCTTCCCTGAAGACCTGAAG-3’ (forward)  5’-CCAGACGGAAACCATAGTCG-3’ (reverse)  Exons 4-6  5’-CTGGCCCCTCTCATCTTCT-3’ (forward)  5’-ACTGTGCCGAAAAGTCTGCT-3’ (reverse)  Exon 6-9  5’-CCGAGTGGAAGGAAATATGC-3’ (forward)  5’-TGTTTTTCTCTTTGGCTGGG-3’ (reverse)  ***p16 (Cdk2na)***  Coding region  5’-ATGGAGCCCTCTGCGGACG-3’ (forward)  5’-GGGGTGGTCCGCGAAATCC-3’ (reverse)  Bisulphite sequencing primers  ***p16 promoter (450 bp upstream region)***  5’- TTGGTTTATTAGTTTAGGAGATTTA -3’ (forward)  5’- ATACTACTCCAAATACTCCCCTATC-3’ (reverse) |
| --- |
